# Evaluating pre-pregnancy dietary diversity vs. dietary quality scores as predictors of gestational diabetes and hypertensive disorders of pregnancy

**DOI:** 10.1371/journal.pone.0195103

**Published:** 2018-04-03

**Authors:** Selma Kronsteiner-Gicevic, Audrey J. Gaskins, Teresa T. Fung, Bernard Rosner, Deirdre K. Tobias, Sheila Isanaka, Walter C. Willett

**Affiliations:** 1 Department of Nutrition, Harvard T.H. Chan School of Public Health, Boston, Massachusetts, United States of America; 2 Channing Division of Network Medicine, Department of Medicine, Brigham and Women's Hospital, Boston, Massachusetts, United States of America; 3 Department of Nutrition, Simmons College, Boston, Massachusetts, United States of America; 4 Department of Biostatistics, Harvard T.H. Chan School of Public Health, Boston, Massachusetts, United States of America; 5 Division of Preventive Medicine, Brigham and Women's Hospital, Boston, Massachusetts, United States of America; 6 Department of Global Health and Population, Harvard T.H. Chan School of Public Health, Boston, Massachusetts, United States of America; 7 Department of Epidemiology, Harvard T.H. Chan School of Public Health, Boston, Massachusetts, United States of America; University of Missouri Columbia, UNITED STATES

## Abstract

**Background:**

Dietary diversity scores (DDS) are considered as metrics for monitoring the implementation of the UN’s Sustainable Development Goals, but they need to be rigorously evaluated.

**Objective:**

To examine two DDS, the Food Groups Index (FGI), and the Minimum Dietary Diversity-Women (MDD-W), alongside two dietary quality scores, the Alternate Healthy Eating Index (AHEI-2010) and the Prime Diet Quality Score (PDQS), with risks of gestational diabetes mellitus (GDM) and hypertensive disorders of pregnancy (HDPs).

**Design:**

The analysis included 21,312 (GDM) and 19,917 (HDPs) singleton births reported in the Nurses’ Health Study II cohort (1991–2001), among women without major chronic disease or GDM/HDPs. Scores were derived using prepregnancy diet collected by a comprehensive food frequency questionnaire. Multivariable models were utilized to calculate relative risks (RR) and confidence intervals (95%CIs).

**Results:**

Incident GDM (n = 916) and HDPs (n = 1,421) were reported. The MDD-W and FGI were not associated with risk of GDM or HDPs, but the AHEI-2010 and PDQS were associated with a lower risk of GDM and marginally lower risk of HDP. The RR’s of GDM comparing the highest vs. lowest quintiles were 1.00 (95%CI: 0.79, 1.27; p-trend = 0.82) for MDD-W, 0.96 (95%CI: 0.76, 1.22; p-trend = 0.88) for FGI, 0.63 (95%CI: 0.50, 0.81; p-trend <0.0001) for the AHEI-2010 and 0.68 (95%CI: 0.54, 0.86; p-trend = 0.003) for the PDQS. Similarly, the RR’s of HDPs were 0.92 (95%CI: 0.75, 1.12, p-trend = 0.94) for MDD-W, 0.97 (95%CI: 0.79, 1.17; p-trend = 0.83) for FGI, 0.84 (95%CI: 0.70, 1.02; p-trend = 0.07) for AHEI-2010 and 0.89 (95%CI: 0.74, 1.09; p-trend = 0.07) for PDQS.

**Conclusions:**

MDD-W and FGI did not predict the risk of GDM and HDPs. These DDS should not be widely used as metrics for achieving dietary goals in their present form. The Prime Diet Quality Score warrants further testing as a promising measure of a sustainable and healthy diet on a global scale.

## Introduction

Dietary diversity scores (DDS), originally developed for assessing nutrient and energy intake adequacy among women and small children in under-resourced settings [1, 2] have been proposed [3–5] as indicators for monitoring the implementation of the UN’s Sustainable Development Goals (SDGs) globally, specifically for Goal 2, that aims to globally ‘reduce all forms of malnutrition by 2030’. These scores, the Minimum Dietary Diversity for Women (MDD-W) and the Food Diversity Index (FGI), consist of ten (MDD-W) and eight (FGI) food groups, including sources of starch (e.g. grains, roots, tubers), animal protein (e.g. flesh and organ meat), dairy and fat (all types), as well as β-carotene-rich and other types of fruits and vegetables, legumes, nuts and eggs. Ideally such an index could also serve as a global dietary guidance tool. However, this is potentially problematic as high consumption of some of their components has been associated with adverse cardiometabolic outcomes [6, 7] and high greenhouse gas production, deforestation, and loss of arable land [8–10]. These potential consequences are inconsistent with the SDG’s Goal 3 to reduce mortality from non-communicable diseases, and the Goals 13–15 to reduce greenhouse gas emission, biodiversity, and arable land loss.

Conceptually, dietary diversity can be a confusing term as it has not been formally defined [11]. It may send a wrong public health message as ‘eating everything in moderation’ does not necessarily equal to adhering to a healthy diet [11–13]. And while dietary diversity may be a way to improve nutrient and energy intake among young children and women in low-income countries [14, 15], it fails to predict obesity [16] or coronary heart disease (CHD) [17] among adult populations. Therefore, any metrics used globally would primarily need to put an emphasis on the quality of diet, rather than merely on the diversity of dietary intake [5], and should undergo rigorous evaluation in relation to a range of health outcomes, in different country settings and across different populations before implemented broadly. One alternative index, the Prime Diet Quality Score (PDQS) was recently developed as a way to characterize diet quality globally and was associated with a lower risk of CHD in a large US population [17]. Whether this diet quality score is useful to predict other adverse health outcomes, including common pregnancy complications, remains to be determined.

Gestational diabetes (GDM) and hypertensive disorders of pregnancy (HDPs) are common pregnancy complications; globally, GDM rates have increased steeply over recent decades, and are projected to rise further in low and medium income countries (LMICs) [18]. HDPs affect 5–18% pregnancies worldwide [19, 20], pose a major cause of maternal death, and are a significant risk factor for fetal death and a range of adverse neonatal and long-term metabolic outcomes for both mother and child [21–26]. Studies of diet-GDM relationships suggest that high intakes of red and processed meats, saturated fats, refined grains, sweets, high fat dairy, and fried foods are associated with a significantly elevated risk of GDM [27–29]. While diet-HDPs associations are less clear [30], some studies indicate that higher calcium, magnesium, and long-chain n-3 fatty acids intakes as well as stronger adherence to a Mediterranean diet may play a role in reducing the risk [29, 31, 32], and that some traditional and Western-style diets are associated with a higher risk of HDPs [33, 34]. Given the high prevalence of HDPs and an expected rise of GDM in LMICs as they undergo nutrition and epidemiologic transitions, the use of simple tools to monitor risk factors for these conditions, including diet diversity scores as a multipurpose diet quality measurement must be carefully evaluated in both high, middle and low-income country settings. This study aimed to examine associations of diet diversity and diet quality scores with risk of GDM and HDPs in a large cohort of US women, hypothesizing that DDS are not associated with these outcomes, whereas higher diet quality scores are associated with a lower risk.

## Methods

The study protocol was approved by the institutional review boards of Brigham and Women’s Hospital and the Harvard T.H. Chan School of Public Health; completion of the self-administered questionnaire was considered to imply informed consent.

### Study design and population

The Nurses’ Health Study II (NHS II) is a prospective cohort established in 1989 that includes 116,671 female registered U.S. nurses, aged 24–44 at baseline. Participants completed self-administered questionnaires on their medical history and lifestyle factors at enrollment, as well as on biennial follow-up questionnaires to update this information and disease outcomes. Dietary intake was assessed every four years using a self-administered food frequency questionnaire (FFQ). For the present analysis, we included all women who reported a singleton live birth from 1991 to 2001 (n = 41,101). We excluded women with a history of chronic disease (type 2 diabetes, cardiovascular disease, or cancer) (n = 3,221) or hypertension or HDPs (in the HDP analysis only) (n = 11,174) prior to pregnancy, a history of GDM (for the GDM analysis) (n = 4,120), or who had a missing or incomplete prepregnancy FFQ (n = 19,842). Women contributed eligible pregnancies until a first diagnosis of GDM or HDP or until the end of follow-up.

### Exposure assessment

To construct the individual prepregnancy dietary scores/indices, we used data collected by a validated 131-item semi-quantitative FFQ distributed to participants every four years starting in 1991 [35, 36]. The FFQ asked participants how often, on average, they had consumed the specified amount of each food or beverage during the past year, offering nine possible responses, ranging from ‘never/once a month’ to ‘6 or more times a day’. A year-specific food composition database created for this study from the USDA database and other sources [37] was used to convert food intake into nutrient intakes. FFQs with calorie intakes <800 kcal/day or >3,500kcal/day and those with more than 70 items missing were excluded from the analysis. The scores computed for all FFQ cycles before a given pregnancy were combined to calculate a cumulative average intake. This provides the best assessment of long-term intake by reducing measurement error and the impact of intra-person variation. *S2 Table* provides a visual comparison of these four scores.

#### Measures of dietary diversity

MDD-W [1] is a 10-item food-based indicator, developed specifically to measure nutrient intake adequacy among women living in under-resourced settings and in developing countries. It includes the following food groups: 1) starchy staples, 2) pulses, 3) nuts and seeds, 4) dairy, 5) meat, poultry and fish, 6) eggs, 7) dark green leafy vegetables, 8) other vitamin-A rich fruits and vegetables, 9) other vegetables, and 10) other fruits. Based on the assumption of dietary diversity being a proxy for dietary quality, it allocates one point for each of the ten food groups consumed over the last 24 hours and zero points otherwise. MDD-W scores range from 0 to 10.

FGI [2] is a similar indicator to MDD-W, developed to measure nutrient intake adequacy among children in under-resourced settings, and in developing countries. The FGI consists of eight items: 1) starchy staples, 2) legumes and nuts, 3) dairy, 4) flesh foods (meat, fish, poultry and liver/organ meats), 5) eggs, 6) vitamin-A rich fruits and vegetables, 7) other fruits and vegetables, and 8) fats and oils. It awards one point for each food group consumed over the past 24 hours (and zero points otherwise), using a 10g cutoff per food group for the first seven groups, and a 1g cutoff for fats and oils. FGI has a range from 0 to 8. Since both scores were based on 24-hr recall data, we developed a revised scoring algorithm for use with FFQ data [17], awarding a point for each food group consumed at least once/day.

#### Measures of dietary quality

Alternate Healthy Eating Index 2010 (AHEI-2010) [38] is an 11-unit, combined nutrient- and food-based score. Points are awarded for intake of each item on a scale from 0 (poorest) to 10 (highest). Higher intake of vegetables (excluding potatoes), fruits, whole grains, nuts and legumes, long chain (n-3) fatty acids, polyunsaturated fats (PUFAs), and moderate alcohol is scored positively, while higher intake of sugar-sweetened beverages and fruit juice, red and processed meats, *trans* fat, and sodium is scored in reverse. The AHEI-2010 ranges from 0 to 110.

The Prime Diet Quality Score (PDQS) [17] is a 21-unit food-based score developed using a modified PrimeScreen questionnaire [39]. It contains 14 “healthy” food groups (dark green leafy vegetables, cruciferous vegetables, carrots, other vegetables, citrus fruits, other fruits, legumes, nuts & seeds, poultry, fish, eggs, whole grains, low fat dairy, and liquid vegetable oils) and 7 “unhealthy” food groups (red meat as a main dish, processed meat, potatoes, refined grains & baked goods, sugar-sweetened beverages, fried foods eaten away from home, and sweets & ice cream). Scores are allocated according to consumption frequency (healthy foods: 0 points for 0–1 servings/week, 1 point for 2–3 servings/week and 2 points for 4+ servings/week, with a reversed scoring for unhealthy foods). PDQS has a range 0–42.

### Outcomes’ assessment

In this analysis GDM and HDPs were self-reported on each biennial questionnaire from 1989 through 2001. In cases where there were more than one singleton pregnancies within a two-year cycle and GDM or HDPs were also reported in the same questionnaire cycle, GDM/HDP status was attributed to the first pregnancy. These outcomes were shown to be accurately reported in separate validation studies, with 94% [40] and 89% (Stuart et al., 2017, unpublished results) of self-reports confirmed by medical records, for the GDM and HDPs, respectfully. Pregnancy-related elevated blood pressure and preeclampsia/toxemia are combined into a single outcome for the purpose of this study. Although the International Society for the Study of Hypertension in Pregnancy (ISSHP) [41] classification of HDPs includes ‘chronic hypertension’, this study only focused on those pregnancies without history of chronic hypertension or HDPs.

### Covariates’ assessment

Race/ethnicity, parental history of diabetes and hypertension were assessed at baseline, while for the time-varying covariates the most recent data prior to each pregnancy from the preceding biennial questionnaire were used. Age was computed from the participant’s date of birth to the date of each questionnaire return. Baseline height and updated body weight were used to calculate prepregnancy body mass index (BMI; kg/m^2^). Total physical activity was ascertained in 1991, 1997 and 2001 using a previously validated questionnaire [42], from which MET-hours per week were calculated. Smoking status, alcohol consumption, sedentary behavior, parity, and dietary supplement use were self-reported at baseline and updated every two years thereafter.

### Statistical analysis

Differences in baseline characteristics in 1991 between women in the top (Q5) vs. those in the lowest (Q1) quintile of prepregnancy dietary scores were assessed using chi-square (categorical variables) and Mann-Whitney U tests (continuous variables). Because the scores were on different scales and not normally distributed, they were standardized by conversion to probit scores, which were subsequently used as continuous variables (1-SD increase). We computed pairwise Spearman correlations between continuous dietary scores to evaluate their similarity at baseline. Dietary scores were evaluated both as a continuous and categorical (quintiles) exposures in logistic regression models with generalized estimating equations, with an exchangeable working correlation structure to account for correlated outcomes between pregnancies. We conducted tests for linear trends across quintiles by modeling the median in each quintile as a continuous variable. The models were initially adjusted for age and race (model 1), and then further adjusted (model 2) for parity/nulliparity, smoking status, physical activity, sedentary behavior, pre-pregnancy alcohol intake (all but AHEI-2010 as this is part of the score), family history of diabetes, family history of hypertension, multivitamin use, and GDM in the past or in the current pregnancy (only HDP models). Because the AHEI-2010 was originally developed using the usual intake data obtained from FFQs, AHEI-2010 models were also adjusted for the total caloric intake, while the other models were not because total caloric intake would not be available if these scores were assessed with a simple set of questions. Finally, all models were also adjusted for pre-pregnancy BMI (model 3). Categorical covariates included indicator variables for missing values. We directly compared the dietary pattern scores in relation to each outcome by including two scores (as probit scores) in a single model using PROC GENMOD procedure in SAS followed by the ESTIMATE statement to obtain p-values for differences.

In a secondary analysis, we evaluated whether there was effect modification by pre-pregnancy BMI (BMI>25 vs. BMI≤25), pre-pregnancy smoking status (current vs. former/never), age (age>35 vs. age≤35), nulliparity (yes/no) or family history of diabetes/hypertension (yes/no) by including a multiplicative interaction term in the models. We also examined the association of individual score components with risk of GDM and HDPs, adjusting for the others, to identify the contributions of each. Several sensitivity analyses were conducted to assess the robustness of our findings. We derived exposure only from the most recent pre-pregnancy questionnaire to compare with the main findings. Further, we restricted our analysis by excluding those FFQs filled in during pregnancy (~20% of pregnancies). Also, we adjusted for the total energy intake in the MDD-W, FGI and PDQS models. Finally, we also scored the FGI using ‘ at least 2–4 times a week’ for 1 point and compared the results to those obtained from ‘at least once a day’ for 1 point. All analyses were conducted using SAS version 9.4 (Cary, NC, USA).

## Results

During ten years of follow-up, 15,214 (GDM) women and 14,339 (HDPs) women contributed 21,312 (GDM) and 19,917 (HDPs) eligible singleton live births to the analysis, reporting 916 GDM and 1,421 HDP events. At baseline, the women with higher dietary diversity and quality scores were, on average, older and more physically active compared to those scoring lower. Women with high dietary diversity scores also had higher BMI and consumed more refined grains, potatoes, red meat, trans fat, and had higher proportion of never smokers compared to those in the lowest quintile. For the dietary quality scores, associations with these variables were in the opposite direction. (**Table 1** for GDM and **S1 Table** for HDPs). The Spearman correlations (**S3 Table**) between scores were the highest between the two diversity scores (Spearman r = 0.71) and the two diet quality scores (Spearman r = 0.68), and much lower between the two different types of scores (Spearman r = 0.04 between AHEI-2010 and FGI and r = 0.27 between AHEI-2010 and MDD-W; r = 0.36 between PDQS and FGI, r = 0.61 between PDQS and MDD-W). All correlations were statistically significant (p<0.0001).

**Table 1:** Pre-pregnancy characteristics by quintiles of dietary pattern scores (GDM)^a^.

	*Dietary diversity scores*				*Dietary quality scores*			
	MDD-W		FGI		AHEI-2010		PDQS	
	Q1 (n = 1,618)	Q5 (n = 2,897)	Q1 (n = 2,080)	Q5 (n = 2,763)	Q1(n = 3,068)	Q5 (n = 3,012)	Q1 (n = 2,942)	Q5 (n = 2,714)
Diet score	2^b^	6	3	6	34	65	14	28
Age (y)	31.6±3.3^c^	32.4±3.2*	32.0±3.3	32.3±3.3*	31.3±3.1	32.7±3.3*	31.2±3.1	32.7±3.3*
White (%)	92	94*	91	95*	93	94*	91	94*
Nulliparity (%)	49	38*	53	30*	30	53*	40	44*
BMI (kg/m2)	23.1±4.4	23.6±4.2*	22.8±4.0	23.8±4.3*	24.0±4.9	22.8±3.5*	23.7±4.9	23.2±3.9*
Physical activity (MET-h/wk)	18.2±25.3	30.6±34.1*	23.0±29.6	27.2±31.8*	16.3±21.9	33.6±36.7*	16.2±23.3	32.6±34.9*
Vegetables (servings/d)	1.1±0.6	4.6±2.1*	1.7±1.1	4.0±2.1*	1.6±1.0	3.8±2.1*	1.3±0.8	4.3±2.1*
Fruit (servings/d)	0.8±0.6	3.0±1.7*	1.4±1.2	2.9±1.7*	1.7±1.2	2.4±1.7*	1.1±1.0	3.1±1.7*
Grains, tubers, white roots (serv/d)	2.1±1.1	3.8±1.5*	2.4±1.3	4.5±1.6*	3.6±1.5	3.5±1.6*	3.1±1.4	4.0±1.7*
Refined grains (servings/d)	1.1±0.8	1.7±0.9*	1.1±0.8	1.7±1.0*	1.7±1.0	1.3±0.8*	1.5±1.0	1.5±0.8*
Potatoes (servings/d)	0.3±0.2	0.4±0.3*	0.2±0.2	0.4±0.3*	0.5±0.3	0.3±0.2*	0.5±0.3	0.3±0.2*
Nuts (servings/d)	0.1±0.2	0.4±0.5*	0.1±0.2	0.5±0.5*	0.2±0.2	0.3±0.5*	0.2±0.2	0.4±0.4*
Legumes (servings/d)	0.2±0.2	0.6±0.5*	0.2±0.2	0.6±0.4*	0.3±0.2	0.5±0.4*	0.2±0.2	0.4±0.4*
Poultry (servings/d)	0.3±0.2	0.7±0.4*	0.3±0.2	0.7±0.4*	0.5±0.3	0.6±0.4*	0.3±0.2	0.6±0.3*
Fish (servings/d)	0.2±0.2	0.4±0.3*	0.2±0.2	0.4±0.3*	0.2±0.2	0.4±0.3*	0.1±0.1	0.4±0.3*
Eggs (servings/d)	0.1±0.1	0.2±0.2*	0.1±0.1	0.2±0.3*	0.2±0.2	0.1±0.2*	0.2±0.2	0.2±0.2*
Red meat (servings/d)	0.5±0.4	0.8±0.5*	0.4±0.3	0.8±0.5*	1.4±0.7	0.4±0.4*	0.9±0.6	0.4±0.4*
Low-fat dairy (servings/d)	0.6±0.9	1.7±1.2*	0.8±0.9	1.8±1.2*	1.4±1.3	1.2±1.1*	0.8±1.1	1.7±1.2*
Alcohol (g/d)	2.8±5.8	3.4±5.3*	2.8±5.5	3.1±5.3*	2.0±5.6	4.3±4.8*	2.5±5.3	3.6±5.5*
Total energy (kcal/d)	1258±357	2245±512*	1269±340	2317±492*	2017±517	1730±519*	1709±535	2019±531*
Carbohydrate (% of energy/d)	50±8	52±6*	53±9	51±6*	50±7	52±8*	49±8	53±7*
Protein (% of energy/d)	18±4	20±3*	18±4	20±3*	18±3	20±4*	18±3	20±3*
Total fat (% of energy/d)	32±6	30±5*	29±6	31±5*	33±5	28±5*	34±6	28±5*
MUFA	*12±3*	*11±2* *	*11±3*	*12±2* *	*13±2*	*10±2* *	*13±2*	*10±2* *
SFA	*12±3*	*10±2* *	*11±3*	*11±2* *	*12±2*	*10±2* *	*13±2*	*10±2* *
Animal fat	*17±5*	*16±4* *	*16±5*	*17±4* *	*20±4*	*14±4* *	*19±5*	*15±4* *
Trans Fat	*2*.*7±1*.*3*	*3*.*3±1*.5*	*2*.*2±1*.*1*	*3*.*7±1*.5*	*4*.*3±1*.*7*	*2*.*3±1*.0*	*3*.*9±1*.*7*	*2*.*6±1*.2*
Glycemic index	55±4	53±3*	54±4	54±3*	56±3	52±3*	56±3	52±3*
Glycemic load	87±35	155±45*	92±36	159±45*	141±45	119±45*	119±47	141±45*
Smoking status (%)								
Never	*69*	*71*	*71*	*73*	*75*	*66* *	*70*	*69*
Ever	*31*	*29*	*29*	*27*	*25*	*34* *	*30*	*31*
Par. history of diabetes (%)	11	12	11	11	11	10	11	10
Par. history of hypertension (%)	49	49	48	47	49	47	49	47

^a^ Higher scores indicate greater dietary diversity/quality. PDQS, Prime Diet Quality score; MDD-W, Minimum Dietary Diversity–Women; FGI, Food Group Index; AHEI-2010, Alternate Healthy Eating Score; MET-h, metabolic equivalent of task-hours; Q, quintile. N = 15,214

^b^ Score median.

^c^ Mean±SD (all such values).

* P<0.05 from a chi-square test for categorical variables or Mann-Whitney Test for continuous variables comparing values in Q1 vs. Q5.

Neither of the two dietary diversity scores was associated with incidence of GDM or HDPs; however, both dietary quality scores were related to significantly lower risk of GDM and marginally lower risk of HDP (**Tables 2 and 3**). In fully adjusted models, the relative risks (aRR) of GDM comparing the highest (Q5) vs. lowest (Q1) quintiles were 0.63 (95%CI: 0.50, 0.81; p-trend <0.0001) for the AHEI-2010 and 0.68 (95%CI: 0.54, 0.86; p-trend = 0.003) for the PDQS. The aRRs for HDPs for similar comparisons were somewhat weaker, 0.84 (95%CI: 0.70, 1.02; p-trend 0.07) for AHEI-2010 and 0.89 (95%CI: 0.74, 1.09; p-trend 0.07) for PDQS. However, the models that did not adjust for BMI (models 2) showed stronger associations between dietary quality scores and the risk of HDPs; aRRs were 0.77 (95%CI: 0.64, 0.93; p-trend 0.005) for AHEI-2010 and 0.88 (95%CI 0.73, 1.07; p-trend 0.05) for PDQS. When modeled as a continuous variable per 1-SD increase, aRRs for GDM were 0.85 (95%CI: 0.72, 0.92) for AHEI-2010 and 0.90 (0.83, 0.96) for PDQS; for HDPs, aRRs were 0.96 (95%CI: 0.90, 1.01) for AHEI-2010 and 0.93 (95%CI: 0.88, 0.99) for PDQS.

**Table 2:** Quintiles of pre-pregnancy dietary diversity/quality scores and GDM risk^a^.

	Q1	Q2	Q3	Q4	Q5	P-trend^f^	Continuous exposure^g^
**MDD-W**							
GDM/pregnancies	132/2824	166/3370	223/5599	220/5325	175/4194		
Model 1^b^	1.0	1.08^e^ (0.86, 1.37)	0.86 (0.69, 1.07)	0.91 (0.73, 1.13)	0.88 (0.70, 1.11)	0.10	0.95 (0.88, 1.02)
Model 2^c^	1.0	1.11 (0.87, 1.40)	0.95 (0.76, 1.19)	1.04 (0.83, 1.30)	1.06 (0.84, 1.35)	0.81	1.02 (0.95, 1.10)
Model ^d^	1.0	1.10 (0.86, 1.39)	0.93 (0.75, 1.17)	1.02 (0.81, 1.27)	1.00 (0.79, 1.27)	0.82	0.99 (0.92, 1.07)
**FGI**							
GDM/pregnancies	140/3273	232/5153	48/1278	342/7664	154/3944		
Model 1	1.0	1.09 (0.88, 1.35)	0.82 (0.59, 1.14)	1.11 (0.91, 1.36)	0.92 (0.73, 1.17)	0.59	0.98 (0.91, 1.05)
Model 2	1.0	1.13 (0.91, 1.40)	0.91 (0.65, 1.28)	1.18 (0.96, 1.45)	1.07 (0.84, 1.36)	0.45	1.03 (0.96, 1.11)
Model 3	1.0	1.09 (0.88, 1.36)	0.78 (0.55, 1.09)	1.11 (0.90, 1.36)	0.96 (0.76, 1.22)	0.88	0.99 (0.92, 1.07)
**AHEI-2010**							
GDM/pregnancies	218/4331	227/4607	182/3926	163/4242	126/4206		
Model 1	1.0	0.96 (0.79, 1.16)	0.88 (0.72, 1.08)	0.71 (0.57, 0.87)	0.54 (0.43, 0.67)	<0.0001	0.80 (0.75, 0.86)
Model 2	1.0	0.99 (0.81, 1.20)	0.94 (0.77, 1.17)	0.75 (0.60, 0.93)	0.58 (0.46, 0.74)	<0.0001	0.83 (0.77, 0.89)
Model 3	1.0	1.01 (0.83, 1.22)	0.99 (0.80, 1.23)	0.80 (0.64, 0.99)	0.63 (0.50, 0.81)	<0.0001	0.85 (0.79, 0.92)
**PDQS**							
GDM/pregnancies	218/3916	184/4115	191/4679	188/4492	135/4110		
Model 1	1.0	0.78 (0.63, 0.95)	0.71 (0.58, 0.86)	0.70 (0.57, 0.86)	0.53 (0.42, 0.66)	<0.0001	0.82 (0.76, 0.88)
Model 2	1.0	0.86 (0.70, 1.05)	0.80 (0.65, 0.98)	0.84 (0.68, 1.03)	0.67 (0.53, 0.85)	0.002	0.89 (0.82, 0.95)
Model 3	1.0	0.87 (0.71, 1.07)	0.81 (0.66, 1.00)	0.86 (0.70, 1.06)	0.68 (0.54, 0.86)	0.003	0.90 (0.83, 0.96)

^a^ Generalized estimating equations (GEE) logistic regression was used to approximate RRs and 95%CIs; higher scores indicate greater dietary diversity/quality. PDQS, Prime Dietary Quality score; MDD-W, Minimum Dietary Diversity–Women; FGI, Food Group Index; AHEI-2010, Alternate Healthy Eating Score; MET-h, metabolic equivalent of task-hours; Q, quintile. N = 21,312 pregnancies, events = 916.

^b^ Model 1: adjusted for age (<30, 30–34, 35–40, ≥40) and race (Caucasian vs. other).

^c^ Model 2: adjusted as for model 1 plus parity (0, 1, 2, 3, 4+), smoking status (never, former or current), physical activity (in MET-h/wk; quartiles), sedentary time (hours sitting at home/work: 0–1, 2–5, 6–10, 11–20 or ≥21), parental history of type 2 diabetes (yes/no), alcohol intake (g/d: 0, 1–14, or ≥15) for all except for aHEI-2010. AHEI-2010 also adjusted for the total caloric intake.

^d^ Model 3: adjusted as for model 2 plus pre-pregnancy BMI (kg/m^2^, categorical <23, 24–25, 26–27, 28–30, 31–34, or ≥35).

^e^ RR; 95% CI in parentheses (all such values).

^f^ Quintile medians were fitted in a multivariate model to estimate P-trend.

^g^ Standardized to probit scores (1-SD).

**Table 3:** Quintiles of pre-pregnancy dietary diversity/quality scores and HDP risk^a^.

	Q1	Q2	Q3	Q4	Q5	P-trend^f^	Continuous exposure^g^
**MDD-W**							
HDP/pregnancies	198/2601	199/3167	405/5213	355/4998	264/3938		
Model 1^b^	1.0	0.81^e^ (0.66, 1.00)	1.02 (0.86, 1.22)	0.93 (0.77, 1.11)	0.87 (0.72, 1.06)	0.47	0.96 (0.90, 1.02)
Model 2^c^	1.0	0.84 (0.68, 1.03)	1.13 (0.95, 1.36)	1.05 (0.87, 1.27)	0.99 (0.82, 1.21)	0.36	1.01 (0.95, 1.08)
Model 3^d^	1.0	0.82 (0.66, 1.01)	1.10 (0.92, 1.32)	1.01 (0.84, 1.22)	0.92 (0.75, 1.12)	0.94	0.98 (0.92, 1.04)
**FGI**							
HDP/pregnancies	225/3028	334/4828	82/1179	531/7218	249/3664		
Model 1	1.0	0.93 (0.78, 1.11)	0.93 (0.71, 1.20)	0.99 (0.84, 1.17)	0.91 (0.75, 1.10)	0.58	0.97 (0.91, 1.03)
Model 2	1.0	0.96 (0.80, 1.15)	1.05 (0.80, 1.37)	1.10 (0.93, 1.30)	1. 09 (0.90, 1.32)	0.13	1.04 (0.97, 1.10)
Model 3	1.0	0.93 (0.78, 1.11)	0.90 (0.68, 1.18)	1.03 (0.87, 1.21)	0.97 (0. 79, 1.17)	0.83	0.99 (0.93, 1.06)
**AHEI-2010**							
HDP/pregnancies	300/4018	319/4275	259/3676	286/3973	257/3975		
Model 1	1.0	1.00 (0.85, 1.18)	0.94 (0.79, 1.12)	0.95 (0.80, 1.13)	0.84 (0.71, 1.01)	0.05	0.95 (0.90, 1.01)
Model 2	1.00	0.97 (0.82, 1.15)	0.93 (0.77, 1.11)	0.91 (0.76, 1.09)	0.77 (0.64, 0.93)	0.005	0.93 (0.87, 0.98)
Model 3	1.00	0.99 (0.84, 1.18)	0.97 (0.81, 1.17)	0.97 (0.81, 1.16)	0.84 (0.70, 1.02)	0.07	0.96 (0.90, 1.01)
**PDQS**							
HDP/pregnancies	273/3624	290/3815	351/4386	263/4213	244/3879		
Model 1	1.0	1.01 (0.85, 1.20)	1.06 (0.90, 1.26)	0.81 (0.68, 0.97)	0.81 (0.68, 0.97)	0.002	0.90 (0.85, 0.95)
Model 2	1.0	1.08 (0.90, 1.28)	1.14 (0.96, 1.35)	0.88 (0.74, 1.06)	0.88 (0.73, 1.07)	0.05	0.93 (0.87, 0.98)
Model 3	1.0	1.09 (0.92, 1.31)	1.16 (0.98, 1.38)	0.89 (0.74, 1.08)	0.89 (0.74, 1.09)	0.07	0.93 (0.88, 0.99)

^a^ Generalized estimating equations (GEE) logistic regression was used to approximate RRs and 95%CIs; higher scores indicate greater dietary diversity/quality. PDQS, Prime Dietary Quality score; MDD-W, Minimum Dietary Diversity–Women; FGI, Food Group Index; AHEI-2010, Alternate Healthy Eating Score; MET-h, metabolic equivalent of task-hours; Q, quintile. N = 19,917 pregnancies, events = 1,421.

^b^ Model 1: adjusted for age (<30, 30–34, 35–40, ≥40) and race (Caucasian vs. other)

^c^ Model 2: adjusted as for model 1 plus nulliparity (yes/no), smoking status (never, former or current), physical activity (in MET-h/wk; quartiles), sedentary time (hours sitting at home/work: 0–1, 2–5, 6–10, 11–20 or ≥21), parental history of hypertension (yes/no), current and past GDM (yes/no), multivitamin use (yes/no), alcohol intake (g/d: 0, 1–14, or ≥15) for all except for aHEI-2010. AHEI-2010 also adjusted for the total caloric intake.

^d^ Model 3: adjusted as for model 2 plus pre-pregnancy BMI (kg/m^2^, categorical <23, 24–25, 26–27, 28–30, 31–34, or ≥35).

^e^ RR; 95% CI in parentheses (all such values).

^f^ Quintile medians were fitted in a multivariate model to estimate P-trend.

^g^ Standardized to probit scores (1-SD).

In the GDM models, tests for heterogeneity showed significant interactions between dietary quality and pre-pregnancy body mass index (BMI), nulliparity and maternal age (**S4 Table**). Higher dietary quality was more strongly associated with a lower risk of GDM among leaner, older (only PDQS), and nulliparous (only AHEI-2010) women. There were no significant interactions between dietary diversity scores and risk of GDM. We also did not find any significant interactions between dietary diversity or quality scores and risk of HDP.

When we compared two dietary scores in a single model **(S5 Table)** we found that PDQS predicted both outcomes similarly to the AHEI-2010 (GDM: p-difference = 0.16, HDPs: p-difference = 0.33) and that both diet quality scores were significantly better predictors of GDM than the two dietary diversity scores (AHEI-2010: p-difference = 0.001 for MDD-W and p-difference = 0.003 for FGI, PDQS: p-difference = 0.002 for MDD-W and p-difference = 0.01 for FGI). Finally, PDQS was a significantly better predictor of HDPs than MDD-W (p-difference = 0.05).

When we examined individual score components in servings/day **(S6 and S7 Tables)**, we found positive associations between animal flesh food intakes (FGI and MDD-W), red meat, processed meat and fried food intakes (PDQS) and linoleic acid intake (AHEI-2010) with GDM, while moderate alcohol intake (AHEI-2010) was associated with a lower risk of GDM. Further, we found positive associations between higher intake of flesh foods (MDD-W and FGI), legumes and nuts (FGI), sodium (AHEI-2010), processed meats, poultry and cruciferous vegetables (PDQS) with the HDPs risk. In addition, after restricting to the most recent pre-pregnancy questionnaire or only FFQs filled out while not pregnant, all relative risk estimates remained similar. However, due to the lower sample size of these sensitivity analyses, the study power was reduced, resulting in wider confidence intervals. And finally, all disease associations for both dietary diversity scores remained nonsignificant when adjusted for total energy intake.

## Discussion

In this large, prospective cohort study, we found that the food-based dietary diversity scores, the FGI and the MDD-W, did not predict GDM or HDPs. However, the dietary quality scores AHEI-2010 and the PDQS were associated with a lower risk of GDM and a slightly lower risk of HDP that was partly accounted for by BMI. The DDSs do not account for the type of carbohydrate and fat and also favorably score all forms of animal protein sources. Therefore, inclusion of refined grains, saturated and trans fatty acids, and red and processed meats, which have been associated with increased risks of several chronic diseases [6] may partly explain the null findings with the DDSs. Another possible explanation is the nature of the setting and the population they were tested in. The DDS may perform better in severely under-resourced areas where dietary diversity indicates achieving energy and nutrient adequacy among undernourished women of reproductive age and young children. In more food-abundant settings, the DDS fail to fully distinguish between healthy and unhealthy food items, and may result in awarding inappropriately high scores to some individuals that consume ‘more of all foods’, including processed foods. Notably, GDM and HDPs are increasing rapidly even in the poorest countries, where access to unhealthy foods is also on the rise, so our findings may be applicable to many women in these countries.

Several other studies have examined the usefulness of DDS across different settings. In a pooled analysis of adult women and men in a high-income country setting, Fung et al. [17] found only a weak inverse association between MDD-W and risk of coronary heart disease (CHD) and no association between FGI and CHD, while PDQS had a strong, inverse association with the outcome. Among HIV-infected adults in Tanzania, Abioye et al. (AI Abioye, Harvard University, personal communication, 2016) found that, while DDS did predict reduced mortality, it did not predict hemoglobin levels or anemia. Similar results for hemoglobin and anemia among Pakistani women were also found by Ali et al.[43]. Finally, a systematic review and meta-analysis of studies of DDS and obesity in LMIC was inconclusive [16]. This growing body of literature warrants caution when utilizing DDS in adult populations and across different populations. While DDS may be reasonable indicators of growth and nutritional status in young children [2, 14, 44, 45], the long-term utility of the DDS as dietary measurement tools for prevention of non-communicable diseases which are increasing rapidly globally [46] remains unclear.

On the other hand, our findings are in line with the literature on the role of overall dietary quality and GDM. In the same population of women, both the Alternative Mediterranean Score (aMED), AHEI and the Dietary Approaches to Stop Hypertension (DASH) scores predicted the risk of GDM comparably [47] and an inverse association was found between adherence to a prudent dietary pattern and risk of GDM and a positive association was found with higher adherence to a Western dietary pattern [48]. Bao et al. [49] also found, in a separate paper from this cohort, that higher intake of animal protein and fat was associated with a higher risk of GDM in this group of women.

While both diet quality scores have a clear inverse relationship with the GDM, this association is somewhat less clear with HDPs. It could be that the long-term pre-pregnancy diet is not as relevant as diet during pregnancy for this outcome. Also, although previous studies suggested a strong influence of non-dietary risk factors, such as high maternal BMI, family history of hypertension, multiple pregnancy and nulliparity on the occurrence of HDPs, the role of diet is inconclusive [30, 50]. After adjusting for pre-pregnancy BMI and family history of hypertension in our study, the relationship between the highest quintile of PDQS and HDPs was no longer significant, but a modest linear trend remained. As the recent literature suggested that a Mediterranean diet was associated with a lower risk of HDPs [32], we also examined whether the (aMED) was more predictive of HDPs in a sensitivity analysis, but the results were also nonsignificant. Data on overall dietary patterns and HDPs are sparse, but recent studies in Australia [32] and Norway [34] suggested that stronger adherence to a Mediterranean or prudent dietary patterns could aid in reducing the occurrence of HDPs. While our study findings also suggested a lower risk, the associations were somewhat weaker. In fact, risk ratios in our models were no longer significant for PDQS and remained borderline significant for AHEI-2010 after adjusting for BMI, indicating a significant role of overweight/obesity in the occurrence of hypertensive disorders of pregnancy. It is possible that inclusion of BMI in the model represents over-control as increased adiposity may be a mediating factor given that the foods included in the PDQS and the AHEI-2010 do predict future weight gain in our cohort [51], and a Mediterranean diet has been shown to reduce weight compared to both low- and high-fat diets [52].

In our cohort, both the AHEI-2010 and the PDQS were more strongly associated with GDM among leaner women. Among overweight and obese women, high adiposity may be a dominant risk factor and therefore low quality diet may not contribute much additional risk. However, this does not imply that good quality diets is not important to women with high BMI; on the contrary, the main goal should be reducing pre-pregnancy BMI to a healthy range by reducing total energy intake and improving dietary quality. Similar to BMI, scoring high on AHEI-2010 appeared to be more strongly associated with GDM risk among nulliparous participants. On the other hand, high dietary quality had a stronger inverse association with GDM among older women, otherwise considered a high-risk group for GDM. If confirmed, this would provide older pregnant women an additional means to maximize the chance of having a healthy pregnancy.

Additionally, we should note that some aspects of DDSs are problematic from the environmental point of view as well; beef and dairy, both scored positively in DDS, are the leading dietary sources of greenhouse gas (GHG) emissions [8], and major contributors to land degradation, water use and biodiversity loss [53]; hence, caution should be exercised when considering inclusion of these dietary components in any dietary metrics.

Our results show that the simple, food-based PDQS predicts both GDM and HDPs comparably to the more complex AHEI-2010. This suggests that a simple food-only tool could be practically used in both field and clinical settings as the AHEI-2010 requires complicated calculations and a food composition database. These findings can also be used to guide development of dietary guidelines in low- and middle- income countries undergoing nutrition transition and projected to experience an increase in these diseases. While it might be tempting to recommend ‘eating all in moderation’ and ‘improve diversity of overall diet’ to improve nutrition status among women and children in the developing world, the fast-shifting environmental and epidemiological situation in these regions calls for caution in doing so. An emphasis on dietary quality or healthy food diversity appears to be the more effective approach.

The main strengths of this study are its prospective design, large sample size and validated measures of both dietary data and the outcomes. We also had regularly updated covariates that allowed for finely adjusting for confounding. However, we cannot rule out potential residual confounding. In addition, data on both lifestyle habits and health status were self-reported, therefore some misreporting is expected. However, both dietary [54] and outcome [40] (JJ Stuart, Harvard University, personal communication, 2017) variables have been shown to have a reasonable degree of validity. We did not purposely assess diet during pregnancy hence we were unable to separately evaluate the associations between diet during pregnancy and the outcomes under study. Our results need to be confirmed in other populations, and diet before and during pregnancy should be examined for association with conditions such as GDM. Finally, the NHS2 cohort consists predominantly of Caucasian, U.S. women, which potentially limits the generalizability of our findings to other racial/ethnic and socio-demographic groups.

In conclusion, among women in an affluent country, we found that neither the MDD-W nor the FGI dietary diversity scores predicted risk of gestational diabetes, whereas the dietary quality scores AHEI-2010 and PDQS did. This suggests that in their present form, these dietary diversity scores should not be widely used as metrics for achieving dietary goals. The PDQS, with its simple score construction, is a promising index for measuring a sustainable, healthy diet on a global scale and warrants further evaluation in relation to other diet-related diseases, particularly in low- and middle- income countries.

## Supporting information

S1 Table Pre-pregnancy characteristics by quintiles of dietary pattern scores (HDPs)^1^. (PDF)

S2 Table Components of the dietary diversity/quality scores. (PDF)

S3 Table Spearman correlation coefficients* of the diet quality scores. (PDF)

S4 Table Multivariable^1^ relative risk (95%CI) of GDM and HDP for 1 SD increase in dietary scores, stratified by major pre-pregnancy risk factors. (PDF)

S5 Table Comparing two scores. (PDF)

S6 Table Associations of individual score components with GDM risk. (PDF)

S7 Table Associations of individual score components with HDP risk. (PDF)
