# Supplementary material for: Evaluating pre-pregnancy dietary diversity vs. dietary quality scores as predictors of gestational diabetes and hypertensive disorders of pregnancy
Source: PLoS One. 2018 Apr 3;13(4):e0195103. doi: 10.1371/journal.pone.0195103 (PMC5882133; doi:10.1371/journal.pone.0195103)
Supplement: S1 Table — (PDF) [file pone.0195103.s001.pdf]

S1 Table: Pre-pregnancy characteristics by quintiles of dietary pattern scores (HDPs)[a]

	Dietary diversity scores				*Dietary quality scores*			
	MDD-W		FGI		AHEI-2010		PDQS	
	Q1(n=1,503)	Q5(n=2,743)	Q1(n=1,943)	Q5(n=2,591)	Q1(n=2,872)	Q5(n=2,875)	Q1(n=2,738)	Q5 (n=2,584)
Diet score	2[b]	6	3	6	34	65	14	28
Age (y)	31.6±3.3[c]	32.4±3.2*	32.0±3.3	32.3±3.2*	31.3±3.1	32.7±3.3*	31.2±3.1	32.7±3.3*
White (%)	1383 (92)	2574 (94)	1754 (90)	2448 (94)*	2658 (93)	2700 (94)*	2486 (91)	2429(94)*
Nulliparity (%)	760 (51)	1047 (39)*	1052 (55)	797 (31)*	876 (31)	1527 (54)*	1125 (42)	1151(45)*
BMI (kg/m2)	23.0±4.3	23.5±4.1*	22.7±3.9	23.6±4.2*	23.9±4.8	22.7±3.4*	23.6±4.8	23.1±3.7*
Physical activity (MET-h/wk)	18.2±25.2	30.8±34.6*	23.2±29.9	27.2±32.1*	16.0±20.7	33.8±37.0*	16.3±23.5	32.8±35.4*
Vegetables (servings/d)	1.1±0.6	4.6±2.1*	1.7±1.1	4.0±2.1*	1.6±1.0	3.8±2.1*	1.3±0.8	4.3±2.1*
Fruit (servings/d)	0.8±0.6	3.1±1.7*	1.4±1.2	2.9±1.7*	1.7±1.2	2.4±1.7*	1.1±0.9	3.1±1.7*
Grains, tubers & white roots (servings/d)	2.1±1.1	3.8±1.6*	2.4±1.3	4.5±1.6*	3.6±1.5	3.5±1.6*	3.1±1.4	4.0±1.6*
Refined grains (servings/d)	1.1±0.8	1.7±0.9*	1.1±0.7	1.7±1.0*	1.7±1.0	1.3±0.8*	1.5±1.0	1.5±0.8*
Potatoes (servings/d)	0.3±0.2	0.4±0.3*	0.2±0.2	0.4±0.3*	0.5±0.3	0.3±0.2*	0.5±0.3	0.3±0.2*
Nuts (servings/d)	0.1±0.2	0.4±0.5*	0.1±0.2	0.5±0.5*	0.2±0.2	0.3±0.5*	0.2±0.2	0.4±0.4*
Legumes (servings/d)	0.2±0.2	0.6±0.5*	0.2±0.2	0.6±0.4*	0.3±0.2	0.5±0.4*	0.2±0.2	0.6±0.5*
Poultry (servings/d)	0.3±0.2	0.7±0.4*	0.3±0.2	0.7±0.3*	0.5±0.3	0.6±0.4*	0.3±0.2	0.6±0.4*
Fish (servings/d)	0.2±0.2	0.4±0.3*	0.2±0.2	0.4±0.3*	0.2±0.2	0.4±0.3*	0.1±0.1	0.4±0.3*
Eggs (servings/d)	0.1±0.1	0.2±0.2*	0.1±0.1	0.2±0.3*	0.2±0.2	0.1±0.2*	0.2±0.2	0.2±0.2*
Red meat (servings/d)	0.5±0.4	0.8±0.5*	0.4±0.3	0.8±0.5*	1.4±0.7	0.4±0.4*	0.9±0.6	0.4±0.4*
Low-fat dairy (servings/d)	0.6±0.9	1.7±1.2*	0.8±0.9	1.8±1.2*	1.4±1.3	1.2±1.1*	0.8±1.1	1.7±1.2*
Alcohol (g/d)	2.8±5.5	3.4±5.3*	2.9±5.5	3.1±5.3	1.9±5.5	4.4±4.9*	2.5±5.2	3.6±5.4*
Total energy (kcal/d)	1261±358	2249±511*	1270±341	2318±492*	2014±517	1732±517*	1709±534	2020±529*
Carbohydrate (% of energy/d)	50±8	52±6*	53±9	51±6*	50±7	52±8*	49±8	53±7*
Protein (% of energy/d)	18±4	20±3*	18±4	20±3*	18±3	20±4*	18±3	20±3*
Total fat (% of energy/d)	32±6	30±5*	29±6	31±5*	33±5	28±5*	34±5	28±5*
MUFA	12±3	11±2*	11±3	11±2*	13±2	10±2*	13±2	10±2*
SFA	12±3	10±2*	11±3	11±2*	12±2	10±2*	12±2	10±2*
Animal fat	17±5	16±4*	16±5	17±4*	20±4	14±4*	19±5	15±4*
Trans Fat	2.7±1.3	3.3±1.5*	2.2±1.1	3.7±1.5*	4.3±1.7	2.3±1.0*	3.9±1.7	2.6±1.2*
Glycemic index	55±4	53±3*	54±4	54±3*	56±3	52±3*	56±3	52±3*
Glycemic load	88±35	155±45*	93±37	160±45*	141±45	119±45*	119±47	141±45*
Smoking status (%)								
Never	69	71	71	73	75	66*	69	69
Ever	31	29	29	27	25	34*	31	31
Parental history of diabetes (%)	10	12	11	11	11	11	11	10
Parental history of hypertension (%)	48	49	47	47	48	47	48	47

[a] Higher scores indicate greater dietary diversity/quality. PDQS, Prime Diet Quality score; MDD-W, Minimum Dietary Diversity – Women; FGI, Food Group Index; AHEI-2010, Alternate Healthy Eating Score; MET-h, metabolic equivalent of task-hours; Q, quintile. N=14,339

[b] Score median.

[c] Mean ± SD (all such values). *P<0.05 from a chi-square test for categorical variables or Mann-Whitney Test for continuous variables comparing values in Q1 vs. Q5.
