# Supplementary material for: Evaluating pre-pregnancy dietary diversity vs. dietary quality scores as predictors of gestational diabetes and hypertensive disorders of pregnancy
Source: PLoS One. 2018 Apr 3;13(4):e0195103. doi: 10.1371/journal.pone.0195103 (PMC5882133; doi:10.1371/journal.pone.0195103)
Supplement: S2 Table — (PDF) [file pone.0195103.s002.pdf]

S2 Table: Components of the dietary diversity/quality scores

	MDD-W	FGI	PDQS	AHEI-2010
Score range	0-10	0-8	0-42	0-110
Scoring criteria	1 point for intake from food group over past 24h	1 point for intake from food group over past 24h	<1 ser/wk=0 pt; 2-3 ser/wk=1 pt; 4≤ ser/wk=2 pt. Reversed scoring for unhealthy foods	Scored from 0-10 on each item based on recommended intake
Vegetables				↑
Fruits				↑ (whole fruit only)
Dark green leafy vegetables	↑		↑	
β-carotene-rich vegetables (and fruits)	↑	↑	↑	
Potatoes			↓	
Cruciferous vegetables			↑	
Other vegetables	↑	↑	↑	
Citrus fruits			↑	
Other fruits	↑	Combined with other vegetables	↑	
Meat, poultry and fish	↑	↑		
Fish			↑	
Poultry			↑	
Red meat			↓	↓
Processed meat			↓	
Legumes		↑	↑	
Pulses	↑			
Nuts (and seeds)	↑	Combined with legumes	↑	↑
Dairy	↑	↑		
Low fat dairy			↑	
Eggs	↑	↑	↑	
Grains, white roots, tubers and plantains	↑	↑		
Whole grains			↑	↑
Refined grains and baked goods			↓	
Sugar sweetened beverages and fruit juice			↓	↓
Sweets and ice cream			↓	
Fried foods eaten away from home			↓	
Liquid vegetable oils		↑	↑	
PUFA (no EPA or DHA)				↑
TFA				↓
Alcohol				moderate
Omega-3 fatty acids (EPA and DHA)				↑
Sodium				↓

MDD-W: Minimum Dietary Diversity – Women; FGI: Food Group Index; PDQS: Prime Diet Quality score; AHEI-2010: Alternate Healthy Eating Index 2010;
Up arrows (↑) denote healthy components and down arrows (↓) denote unhealthy components.
Higher score represents higher dietary quality/dietary diversity. PUFA: polyunsaturated fatty acids; TFA: trans fatty acids; EPA: Eicosapentaenoic acid; DHA: Docosahexaenoic acid.
