# Supplementary material for: Evaluating pre-pregnancy dietary diversity vs. dietary quality scores as predictors of gestational diabetes and hypertensive disorders of pregnancy
Source: PLoS One. 2018 Apr 3;13(4):e0195103. doi: 10.1371/journal.pone.0195103 (PMC5882133; doi:10.1371/journal.pone.0195103)
Supplement: S3 Table — (PDF) [file pone.0195103.s003.pdf]

S3 Table: Spearman correlation coefficients[a] of the diet quality scores

	PDQS	MDD-W	FGI	AHEI-2010
PDQS	-	0.61 (0.62)	0.36 (0.32)	0.68 (0.75)
MDD-W	-	-	0.71 (0.57)	0.27 (0.46)
FGI	-	-	-	0.04 (0.20)

[a]Energy-adjusted values in brackets. All $p<0.0001$, N=15,214 (GDM), 14,339 (HDP)
