# Supplementary material for: Evaluating pre-pregnancy dietary diversity vs. dietary quality scores as predictors of gestational diabetes and hypertensive disorders of pregnancy
Source: PLoS One. 2018 Apr 3;13(4):e0195103. doi: 10.1371/journal.pone.0195103 (PMC5882133; doi:10.1371/journal.pone.0195103)
Supplement: S4 Table — (PDF) [file pone.0195103.s004.pdf]

S4 Table: Multivariable[a] relative risk (95%CI) of GDM and HDP for 1 SD increase in dietary scores, stratified by major pre-pregnancy risk factors

Outcome = GDM	MDD-W	FGI	AHEI-2010	PDQS
**BMI status**				
BMI<25 (events=451)	0.97 (0.88, 1.08)	1.00 (0.90, 1.11)	0.78 (0.71, 0.86)	0.84 (0.76, 0.93)
BMI≥25 (events =465)	1.02 (0.92, 1.14)	0.99 (0.89, 1.11)	0.93 (0.84, 1.03)	0.96 (0.87, 1.06)
p-interaction	0.44	0.85	0.004*	0.03*
**Family history of diabetes**				
No family history (events = 728)	1.00 (0.92, 1.09)	1.01 (0.92, 1.09)	0.88 (0.82, 0.96)	0.92 (0.85, 1.00)
Family history (events =188)	0.97 (0.82, 1.16)	0.97 (0.82, 1.15)	0.74 (0.62, 0.88)	0.80 (0.67, 0.95)
p-interaction	0.59	0.64	0.07	0.09
**Smoking status**				
Non smoker (events =817)	0.99 (0.91, 1.07)	0.99 (0.91, 1.07)	0.86 (0.80, 0.93)	0.90 (0.83, 0.97)
Smoker (events =99)	1.04 (0.82. 1.31)	1.08 (0.83, 1.41)	0.76 (0.60, 0.96)	0.85 (0.69, 1.05)
p-interaction	0.91	0.58	0.29	0.60
**Nulliparity**				
Parous (events = 592)	0.99 (0.90 1.09)	0.95 (0.87, 1.05)	0.92 (0.84, 1.00)	0.92 (0.85, 1.01)
Nulliparous (events =324)	1.00 (0.88, 1.13)	1.08 (0.95, 1.22)	0.75 (0.66, 0.85)	0.84 (0.74, 0.95)
p-interaction	0.99	0.12	0.01*	0.22
**Maternal age**				
Age < 35 (events =368)	1.07 (0.95, 1.21)	1.05 (0.94, 1.18)	0.91 (0.82, 1.02)	0.98 (0.87, 1.09)
Age ≥ 35 (events =548)	0.95 (0.86, 1.04)	0.96 (0.87, 1.06)	0.82 (0.74, 0.90)	0.84 (0.77, 0.93)
p-interaction	0.08	0.42	0.74	0.02*
Outcome= HDP				
**BMI status**				
BMI<25 (events = 748)	1.00 (0.92, 1.08)	1.02 (0.93, 1.11)	0.93 (0.86, 1.01)	0.92 (0.85, 0.99)
BMI≥25 (events = 673)	0.97 (0.88, 1.07)	0.99 (0.90, 1.08)	0.96 (0.88, 1.04)	0.93 (0.85, 1.01)
p-interaction	0.78	0.57	0.36	0.69
**Family history of hypertension**				
No family history (events = 591)	0.94 (0.85, 1.03)	1.00 (0.90, 1.10)	0.93 (0.85, 1.01)	0.91 (0.83, 0.99)
Family history (events = 830)	1.02 (0.94, 1.10)	0.99 (0.91, 1.08)	0.98 (0.90, 1.06)	0.95 (0.88, 1.02)
p-interaction	0.36	0.93	0.54	0.68
**Smoking status**				
Non smoker (events = 1308)	0.97 (0.91, 1.04)	0.98 (0.92, 1.05)	0.96 (0.90, 1.02)	0.93 (0.87, 0.99)
Current smoker (events = 113)	1.10 (0.89, 1.37)	1.13 (0.89, 1.44)	0.93 (0.74, 1.17)	0.93 (0.76, 1.14)
p-interaction	0.20	0.16	0.57	0.91
**Nulliparity**				
Parous (events = 726)	0.97 (0.89, 1.06)	0.99 (0.91, 1.08)	0.96 (0.89, 1.04)	0.93 (0.86, 1.00)
Nulliparous (events =695)	0.99 (0.91, 1.08)	0.99 (0.91, 1.09)	0.96 (0.88, 1.04)	0.94 (0.86, 1.02)
p-interaction	0.77	0.81	0.70	0.98
**Maternal age**				
Age <35 (events=684)	1.00 (0.91, 1.09)	1.06 (0.96, 1.17)	0.96 (0.88, 1.04)	0.97 (0.89, 1.06)
Age ≥ 35 (events=737)	0.97 (0.89, 1.06)	0.94 (0.87, 1.03)	0.96 (0.88, 1.03)	0.90 (0.83, 0.97)
p-interaction	0.78	0.13	0.88	0.31

[a] Adjusted for age, race, BMI, smoking, alcohol (except AHEI-2010) physical activity, sedentary behavior, parity, family history of diabetes (GDM)/hypertension (HDP), past and present pregnancy GDM (only HDP), multivitamin use (only HDP), total energy intake (only AHEI-2010). N=15,214 (GDM), 14,339 (HDP)
