# Supplementary material for: Evaluating pre-pregnancy dietary diversity vs. dietary quality scores as predictors of gestational diabetes and hypertensive disorders of pregnancy
Source: PLoS One. 2018 Apr 3;13(4):e0195103. doi: 10.1371/journal.pone.0195103 (PMC5882133; doi:10.1371/journal.pone.0195103)
Supplement: S5 Table — (PDF) [file pone.0195103.s005.pdf]

S5 Table: comparing two scores

Two scores in a single model		p-difference[a] (GDM)	p-difference[a] (HDPs)
AHEI-2010	MDD-W	0.001[b]	0.44
	FGI	0.003 [b]	0.35
PDQS	MDD-W	0.002 [b]	0.05 [b]
	FGI	0.01 [b]	0.06
AHEI-2010	PDQS	0.16	0.33

[a] Scores standardized to probit scores (1-SD), compared in a single model, testing hypothesis that $\beta1=\beta2$
[b] Statistically significant differences ($p<0.05$)
