# Supplementary material for: Evaluating pre-pregnancy dietary diversity vs. dietary quality scores as predictors of gestational diabetes and hypertensive disorders of pregnancy
Source: PLoS One. 2018 Apr 3;13(4):e0195103. doi: 10.1371/journal.pone.0195103 (PMC5882133; doi:10.1371/journal.pone.0195103)
Supplement: S6 Table — (PDF) [file pone.0195103.s006.pdf]

S6 Table: Associations of individual score components with GDM risk

Score components	RR (95% CI)[a]
MDD-W (1 serving/d)	
Grains, white roots, tubers and plantains	0.99 (0.94, 1.04)
**Meat, fish and poultry**	**1.30 (1.18, 1.43)** [d]
Nuts	0.80 (0.63, 1.02)
Pulses (beans, peas and lentils)	0.90 (0.67, 1.20)
Dairy	0.99 (0.94, 1.05)
Eggs	0.88 (0.64, 1.21)
Dark green leafy vegetables	1.00 (0.88, 1.14)
Other high β-carotene fruits and vegetables	0.97 (0.82, 1.15)
Other fruits[b]	0.98 (0.91, 1.05)
Other vegetables[b]	0.99 (0.92, 1.06)
FGI (1 serving/d)	
Grains, white roots, tubers and plantains	0.98 (0.93, 1.03)
**Flesh foods**	**1.29 (1.17, 1.43)** [d]
Legumes and nuts	0.86 (0.74, 1.01)
Dairy	0.99 (0.93, 1.04)
Eggs	0.85 (0.62, 1.18)
β-carotene fruits and vegetables	1.02 (0.88, 1.19)
Other fruits and vegetables[2]	0.97 (0.94, 1.01)
Added fats and oils	1.05 (0.98, 1.11)
AHEI-2010 (1 serving/d)	
Vegetables	1.00 (0.95, 1.04)
Fruits	0.93 (0.85, 1.02)
Whole grains	1.00 (0.99, 1.00)
Sugar-sweetened beverages and fruit juice[3]	0.96 (0.88, 1.05)
Nuts and legumes	0.91 (0.82, 1.02)
Red/processed meat[c]	1.04 (0.99, 1.10)
Trans fatty acids (g/day, in quintiles)[c]	1.02 (0.84, 1.25)
Long chain (n-3) fatty acids (DHA+EPA) (g/day, in quintiles)	1.08 (0.90, 1.30)
**Polyunsaturated fatty acids (linoleic acid) (g/day, in quintiles)**	**1.26 (1.02, 1.55)** [d]
Sodium (mg/day, in quintiles)[c]	0.97 (0.77, 1.23)
**Alcohol (drinks/day)**	**0.78 (0.62, 0.99)** [d]
PDQS (1 serving/d)	
**Processed meat**[c]	**1.35 (1.06, 1.73)** [d]
**Red meat**[c]	**1.41 (1.17, 1.69)**[d]
Nuts	0.83 (0.65, 1.05)
Legumes	0.90 (0.67, 1.20)
Fish	0.98 (0.70, 1.39)
Poultry	1.06 (0.85, 1.32)
Eggs	0.88 (0.62, 1.25)
Low-fat dairy	1.00 (0.94, 1.07)
Whole grains	0.97 (0.91, 1.05)
Refined grains[c]	1.02 (0.95, 1.10)
Potatoes[c]	0.97 (0.77, 1.22)
Dark green leafy vegetables	1.05 (0.91, 1.22)
Carrots	0.97 (0.81, 1.16)
Cruciferous vegetables	1.02 (0.84, 1.26)
Other vegetables	1.00 (0.93, 1.07)
Citrus fruits	0.95 (0.83, 1.09)
Other fruits	1.00 (0.93, 1.08)
**Sweets**[c]	**0.92 (0.85, 1.00)**[d]
Sugar-sweetened beverages[c]	1.02 (0.94, 1.10)
Liquid fats	0.98 (0.80, 1.18)
**Fried foods away from home**[c]	**1.67 (1.03, 2.71)** [d]

[a] Adjusted for other score components, as well as for age, race, physical activity, smoking, sedentary behavior, BMI, family history of type 2 diabetes, parity, alcohol intake (except for AHEI-2010), total caloric intake (AHEI-2010 only)
[b] Other than high β-carotene fruits and vegetables.
[c] These components are given greater score with decreasing intake.
[d] Statistically significant ($p<0.05$)
