# Supplementary material for: Evaluating pre-pregnancy dietary diversity vs. dietary quality scores as predictors of gestational diabetes and hypertensive disorders of pregnancy
Source: PLoS One. 2018 Apr 3;13(4):e0195103. doi: 10.1371/journal.pone.0195103 (PMC5882133; doi:10.1371/journal.pone.0195103)
Supplement: S7 Table — (PDF) [file pone.0195103.s007.pdf]

S7 Table: Associations of individual score components with HDP risk

Score components	RR (95% CI)[a]
MDD-W (1 serving/d)	
Grains, white roots, tubers and plantains	1.01 (0.97, 1.05)
**Meat, fish and poultry**	**1.18 (1.09, 1.28)** [d]
Nuts	1.09 (0.92, 1.28)
Pulses (beans, peas and lentils)	1.16 (0.94, 1.42)
Dairy	0.97 (0.93, 1.02)
Eggs	1.04 (0.81, 1.35)
Dark green leafy vegetables	0.98 (0.86, 1.11)
Other high β-carotene fruits and vegetables	0.91 (0.78, 1.06)
Other fruits[b]	0.97 (0.92, 1.03)
Other vegetables[b]	0.97 (0.91, 1.03)
FGI (1 serving/d)	
Grains, white roots, tubers and plantains	1.01 (0.97, 1.05)
**Flesh foods**	**1.18 (1.08, 1.28)** [d]
**Legumes and nuts**	**1.12 (1.00, 1.25)**[d]
Dairy	0.97 (0.93, 1.02)
Eggs	1.05 (0.81, 1.36)
β-carotene fruits and vegetables	0.92 (0.80, 1.06)
Other fruits and vegetables[b]	0.97 (0.94, 1.00)
Added fats and oils	0.99 (0.94, 1.05)
AHEI-2010 (1 serving/d)	
Vegetables	0.99 (0.96, 1.03)
Fruits	0.97 (0.90, 1.05)
Whole grains	1.00 (0.99, 1.00)
Sugar-sweetened beverages and fruit juice[c]	0.99 (0.92, 1.06)
Nuts and legumes	1.05 (0.97, 1.15)
Red/processed meat[c]	1.00 (0.95, 1.06)
Trans fatty acids (g/day, in quintiles)[c]	1.12 (0.96, 1.32)
Long chain (n-3) fatty acids (DHA+EPA) (g/day, in quintiles)	1.00 (0.86, 1.16)
Polyunsaturated fatty acids (linoleic acid) (g/day, in quintiles)	0.97 (0.81, 1.16)
**Sodium (mg/day, in quintiles)**[c]	**1.30 (1.08, 1.57)**[d]
Alcohol (drinks/day)	0.97 (0.84, 1.11)
PDQS (1 serving/d)	
**Processed meat**[c]	**1.24 (1.02, 1.51)**[d]
Red meat[c]	1.03 (0.88, 1.20)
Nuts	1.08 (0.92, 1.28)
Legumes	1.12 (0.91, 1.37)
Fish	1.00 (0.76, 1.30)
**Poultry (chicken and turkey with/without skin)**	**1.27 (1.06, 1.52)**[d]
Eggs	1.04 (0.80, 1.35)
Low-fat dairy	0.99 (0.94, 1.05)
Whole grains	1.01 (0.96, 1.07)
Refined grains[c]	1.00 (0.94, 1.06)
Potatoes[c]	1.14 (0.95, 1.38)
Dark green leafy vegetables	0.97 (0.85, 1.11)
Carrots	0.88 (0.74, 1.05)
**Cruciferous vegetables**	**1.21 (1.04, 1.41)**[d]
Other vegetables	0.96 (0.91, 1.02)
Citrus fruits	0.96 (0.84, 1.08)
Other fruits	0.97 (0.91, 1.04)
Sweets[c]	1.01 (0.95, 1.07)
Sugar-sweetened beverages[c]	1.04 (0.98, 1.11)
Liquid fats	0.95 (0.81, 1.10)
Fried foods away from home[c]	1.26 (0.84, 1.88)

[a] Adjusted for other score components, as well as for age, race, physical activity, smoking, sedentary behavior, BMI, family history of type 2 diabetes, parity, alcohol intake (except for AHEI-2010), total caloric intake (AHEI-2010 only)
[b] Other than high β-carotene fruits and vegetables.
[c] These components are given a greater score with decreasing intake.
[d] Statistically significant ($p<0.05$)
